# Ethnic differences in pathways to obtain care, maintain care and engage to Early Intervention Service in Spain

**DOI:** 10.1192/j.eurpsy.2024.366

**Published:** 2024-08-27

**Authors:** V. Sanchez-Gistau, M. J. Algora, A. Cabezas, M. Sole, P. Miro, C. Saez

**Affiliations:** early intervention service, University Hospital Institut pere Mata, reus, Spain

## Abstract

**Introduction:**

Ethnicity and migration have an impact on illness models and consequently how, when and where people seek and obtain care. Early Intervention Psychosis (EIP) teams attend high rates of migrant and ethnic diverse populations but the study of ethnic differences in pathways to obtain and maintain care is still scarce . The most consistent findings are that minorities are less involved with primary services, have a higher risk of being treated in a coercive way and are at higher risk of early disengagement. Despite the increasing migration rates there has been very little investigation in Spain.

**Objectives:**

To investigate ethno-racial differences in pathways to obtain care, adherence and engagement during their first year of follow-up of subjects who start treatment at EIP of Reus, Catalonia, Spain

**Methods:**

***Participants***

This is an observational 12 months follow-up retrospective study including all consecutively subjects with First Episode of Psychosis (FEP) referred to the EIP from January 2015 to January 2019. Visible ethno-racial status was self-reported being grouped as belonging to minority ethno-racial group if they were coded “any other than White regardless of country of origin”. ‘White’ was the majority group

***Study variables:***

At program entry:

Source of referral

At 12- months:

Adherence to the service:

Admissions to in-patient unit

Disengagement

**Results:**

184 FEP subjects (mean age 22.8 years and 66.1% of males) were included. Nearly 31% belonged to a minority ethno-racial group being the Maghrebi (60.4%) followed by the Latin-American (20.1%) the most frequent minority groups. The 81.2% of the minority group were first-generation migrants and 7.5% second generation migrants. The 52.2 % were referred from community services, 18.5% from emergency unit and 29.3% from inpatient unit. At follow up 64.5%, were highly adherent to EIP (> 75% of attended appointments), 16.7% required hospitalization and 11% disengaged. Multivariate analysis showed that the minority group was 2.19 times more likely to be low adherent [(95% CI 0.78-3.17; p=0.03], 2.89 times more likely to be hospitalised [(95% CI 1.20-6.98); p=0.01], and 4 times more likely to disengage from the EIP [(95% CI 1.35-11.90); p= 0.01] during follow-up than the majority group. No group differences were found in pathways to obtain care or in causes of disengagement.

**Conclusions:**

In agreement with previous studies from other countries we found high rates of ethno-racial diversity in the EIP of Reus. In addition, we also found inequalities in the use of services, being minorities more likely to disengage, to be low adherent to the program and at greater risk of hospitalization. On contrary to other studies we did not find significant differences between groups in the source of referral to EIP

**Disclosure of Interest:**

None Declared

